# MiR-29c is downregulated in gastric carcinomas and regulates cell proliferation by targeting RCC2

**DOI:** 10.1186/1476-4598-12-15

**Published:** 2013-02-25

**Authors:** Mitsuhiro Matsuo, Chisato Nakada, Yoshiyuki Tsukamoto, Tsuyoshi Noguchi, Tomohisa Uchida, Naoki Hijiya, Keiko Matsuura, Masatsugu Moriyama

**Affiliations:** 1 Department of Molecular Pathology, Faculty of Medicine, Oita University, Oita, Japan; 2 Center for Community Medicine, Division of Surgery, Faculty of Medicine, Oita University, Oita, Japan

**Keywords:** Gastric carcinoma, miR-29c, RCC2

## Abstract

**Background:**

Previously, using miRNA microarray, we have found that miR-29c is significantly downregulated in advanced gastric carcinoma. In the present study, we investigated whether miR-29c functions as a tumor-suppressor miRNA in gastric carcinoma cells. For this purpose, we verified the downregulation of miR-29c in gastric carcinoma tissues, and assessed the biological effect of miR-29c on gastric carcinoma cells.

**Results:**

In miR-29c-transfected cells, both proliferation and colony formation ability on soft agar were significantly decreased. Although apoptosis was not induced, BrdU incorporation and the proportion of cells positive for phospho-histone H3 (S10) were significantly decreased in miR-29c-transfected cells, indicating that miR-29c may be involved in the regulation of cell proliferation. To explain the mechanism of growth suppression by miR-29c, we explored differentially expressed genes (>2-fold) in miR-29c-transfected cells in comparison with negative control transfected cells using microarray. RCC2, PPIC and CDK6 were commonly downregulated in miR-29c-transfected MKN45, MKN7 and MKN74 cells, and all of the genes harbored miR-29c target sequences in the 3’-UTR of their mRNA. RCC2 and PPIC were actually upregulated in gastric carcinoma tissues, and therefore both were identified as possible targets of miR-29c in gastric carcinoma. To ascertain whether downregulation of RCC2 and/or PPIC is involved in the growth suppression by miR-29c, we transfected siRNAs against RCC2 and PPIC into MKN45 and determined cell viability, the rate of BrdU incorporation, and caspase activity. We found that RCC2-knockdown decreased both cell viability and BrdU incorporation without any increase of caspase activity, while PPIC-knockdown did not, indicating that downregulation of RCC2 may be at least partly responsible for the growth suppression by miR-29c.

**Conclusions:**

Our findings indicate that miR-29c may have tumor-suppressive functions in gastric carcinoma cells, and that its decreased expression may confer a growth advantage on tumor cells via aberrant expression of RCC2.

## Background

Gastric carcinoma is one of the most common malignancies and ranks second in terms of global carcinoma-related mortality [1]. The clinical outcome of gastric cancer has gradually improved, but the prognosis of patients with advanced disease is still disappointing. Although alterations in a large number of oncogenic and tumor-suppressive genes are reportedly implicated in gastric carcinoma, the molecular mechanisms underlying the development of gastric carcinoma are still poorly understood. Identification of these mechanisms is necessary for the development of targeted clinical therapy.

MicroRNA (miRNA) is a small non-coding RNA molecule comprising about 22 nucleotides, which regulates the expression of target genes at the post-transcriptional level [2]. It has been reported that miRNAs are frequently dysregulated in human cancers and play oncogenic or tumor-suppressive roles in carcinoma cells [3-5]. In our previous study, using a miRNA microarray covering 470 human miRNAs (miRBase 9.1), we identified 39 miRNAs that were differentially expressed in gastric carcinoma relative to normal gastric epithelium; 6 of these were significantly downregulated and the other 33 were upregulated [6]. We have also reported that miR-375 is the most down-regulated miRNA, and that its ectopic expression is able to induce apoptosis of gastric carcinoma cells through downregulation of PDK1 and 14-3-3zeta [6], implying a tumor-suppressive role of miR-375 in gastric carcinoma cells. Another miRNA, miR-29c, has also been found to be downregulated next to miR-375 [6]. MiR-29c is the member of the miR-29 family composed of miR-29a, -29b and -29c. Deep sequencing has revealed that this miRNA family is that most highly expressed in gastric tissues [7], and that the read count of miR-29c is the highest among them [7], suggesting that alteration of the miR-29 expression level has an impact on gastric cells. Therefore, we hypothesized that miR-29c could be a candidate tumor-suppressor miRNA in gastric carcinoma, as well as miR-375. In the present study, to test this hypothesis, we attempted to clarify the function of miR-29c in the tumorigenesis and/or progression of gastric cancer.

## Results

### Quantification of miR-29c in gastric carcinoma

**Figure 1:**
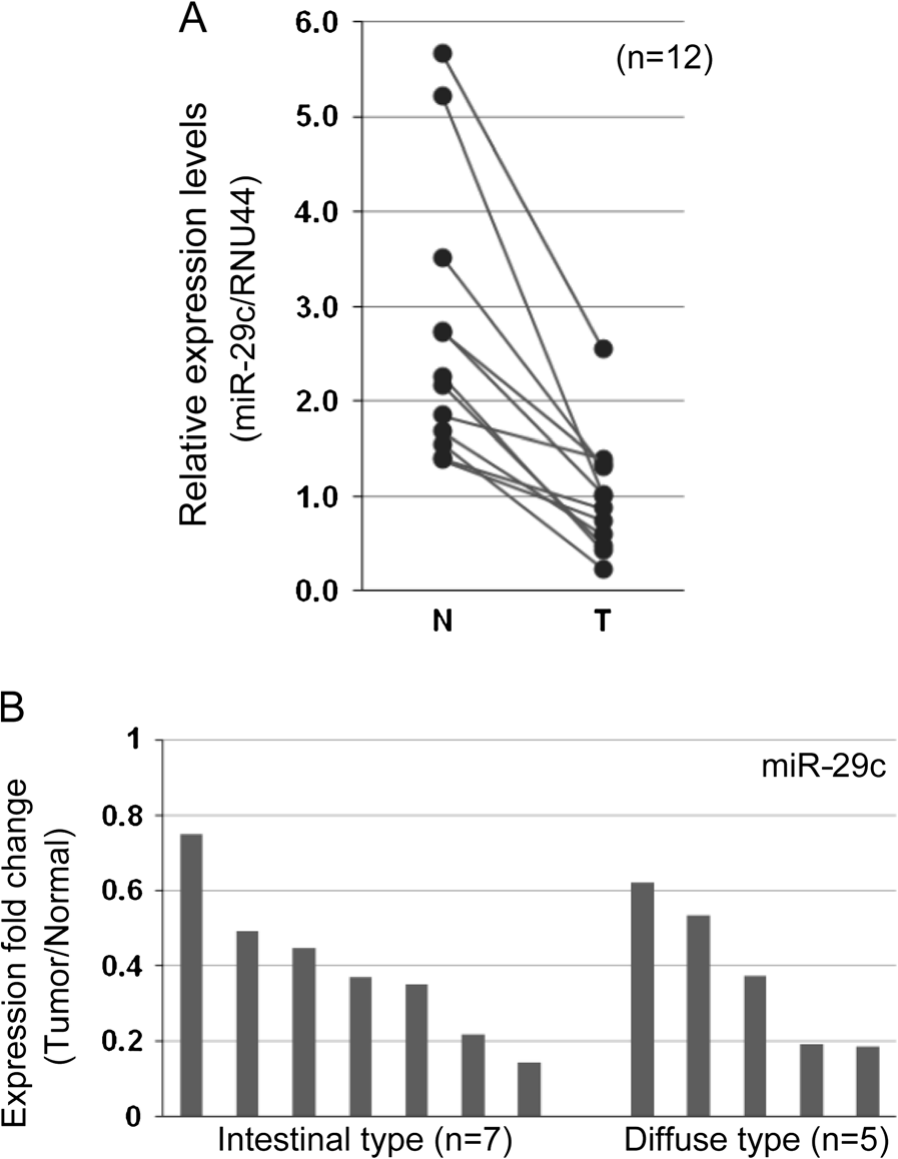
**miR-29c is downregulated in gastric carcinoma tissues.** (**A**) Quantitative RT-PCR was performed using 12 paired samples of gastric carcinoma tissue (T) and adjacent normal tissue (N). Each dot indicates the expression level in an individual case. The Y axis displays the relative expression level of miR-29c normalized by expression of the internal control, RNU44. p = 0.0002. Paired Student’s *t* test. (**B**) The graph demonstrates the fold change in the level of miR-29c expression in the tumor relative to that in normal tissue. Each bar indicates the expression level in an individual case.

To confirm that miR-29c was downregulated in gastric carcinoma, as determined by miRNA microarray [6], we performed quantitative RT-PCR using 12 paired samples of tumor tissue and the adjacent normal epithelium (Additional file 1). Indeed, miR-29c was significantly downregulated in the tumor tissues from all 12 cases (Figure 1A), which comprised 7 intestinal-type and 5 diffuse-type carcinomas (Additional file 1). The degree of miR-29c downregulation was not associated with clinicopathological features such as histological classification, patient age, sex, tumor location, depth and stage (Figure 1B and Additional file 1).

It has been reported that epigenetic modifications such as DNA methylation might influence genome-wide gene expression during tumorigenesis of gastric carcinoma [8-11]. To determine whether epigenetic modification is associated with miR-29c downregulation, we treated MKN45 cells with 5-aza-2’-deoxycytidine and trichostatin A and assessed the subsequent miR-29c expression. As shown in Additional file 2, the expression of miR-29c was not increased by treatment with these reagents, suggesting that epigenetic modifications may not be associated with the downregulation of miR-29c in gastric carcinoma cells.

### Exogenous expression of miR-29c suppresses the proliferation of gastric carcinoma cells

**Figure 2:**
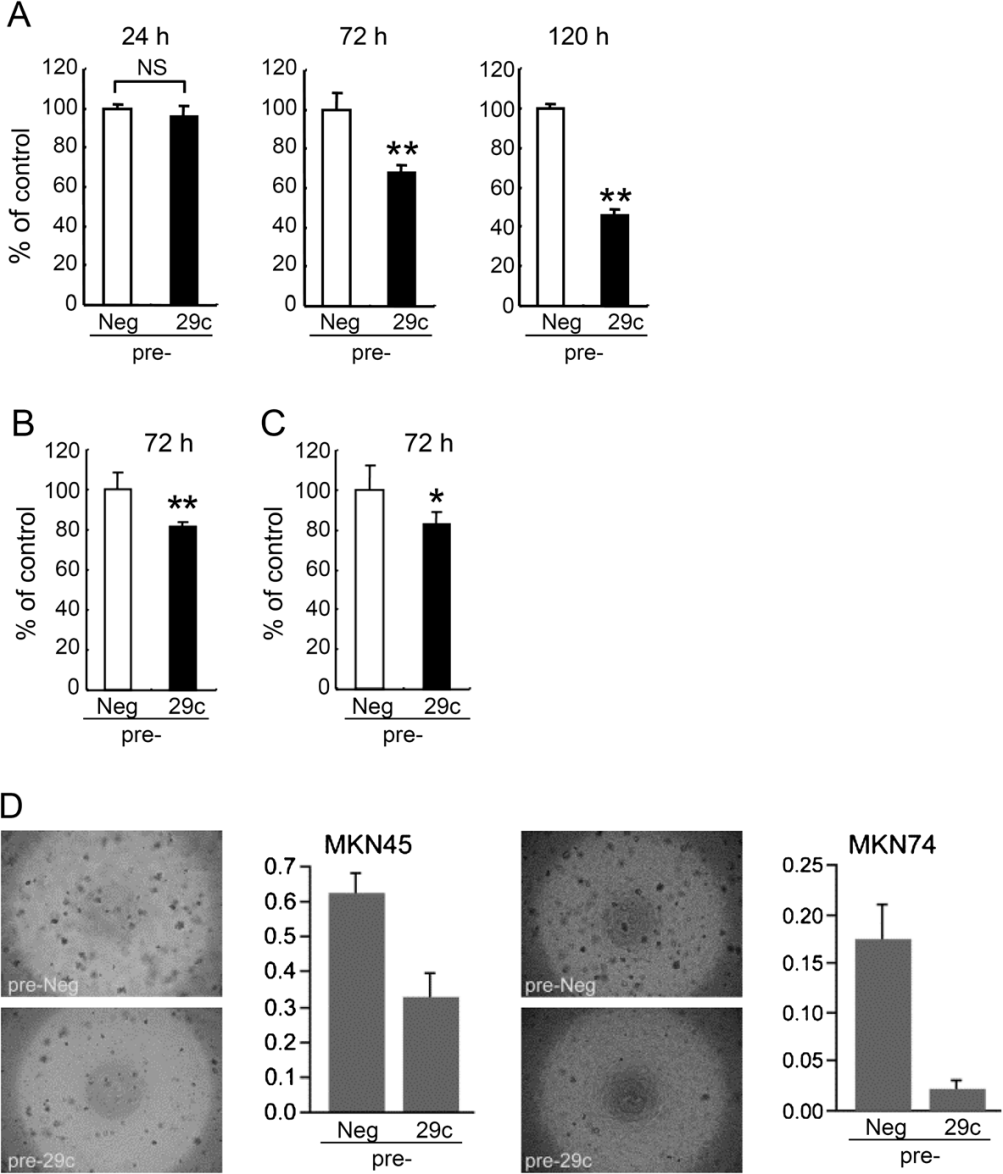
**Cell viability and colony formation efficiency of gastric carcinoma cells on soft agar are decreased by exogenous miR-29c.** Gastric carcinoma cells were transfected with pre-29c or pre-Neg, and then cell viability was determined by MTS assay after the indicated periods. (**A**) MKN45, (**B**) MKN74, (**C**) MKN7. These data are shown as mean ± SD for five wells. NS, not significant. **P <0.01, *P <0.05. (**D**) The graphs show the efficiency of colony formation on soft agar. The Y axis displays the relative fluorescence units (485 nm/538 nm). Data are shown as mean ± SD for four wells. MKN45, *P* =0.006. MKN74, *P* =0.002.

To investigate the biological function of miR-29c in gastric carcinoma, we first assessed the effects of its expression on cell viability using the MTS assay. We transfected precursor miR-29c (pre-29c) or negative control (pre-Neg) into three gastric carcinoma cell lines – MKN45, MKN74 and MKN7 – which showed very low levels of miR-29c expression in comparison with normal epithelial tissues (data not shown), and found that cell viabilities in pre-29c-transfected cells were decreased in all of 3 cell lines tested (Figure 2). The suppression of proliferation occurred in a time-dependent manner in MKN45 (Figure 2A), and similar results were obtained in MKN74 and MKN7 cells (Figures 2B and 2C). MiR-29c also reduced the ability of MKN45 and MKN74 to form colonies in soft agar (Figure 2D). These results suggest that miR-29c may have tumor-suppressive functions in gastric carcinoma cells.

**Figure 3:**
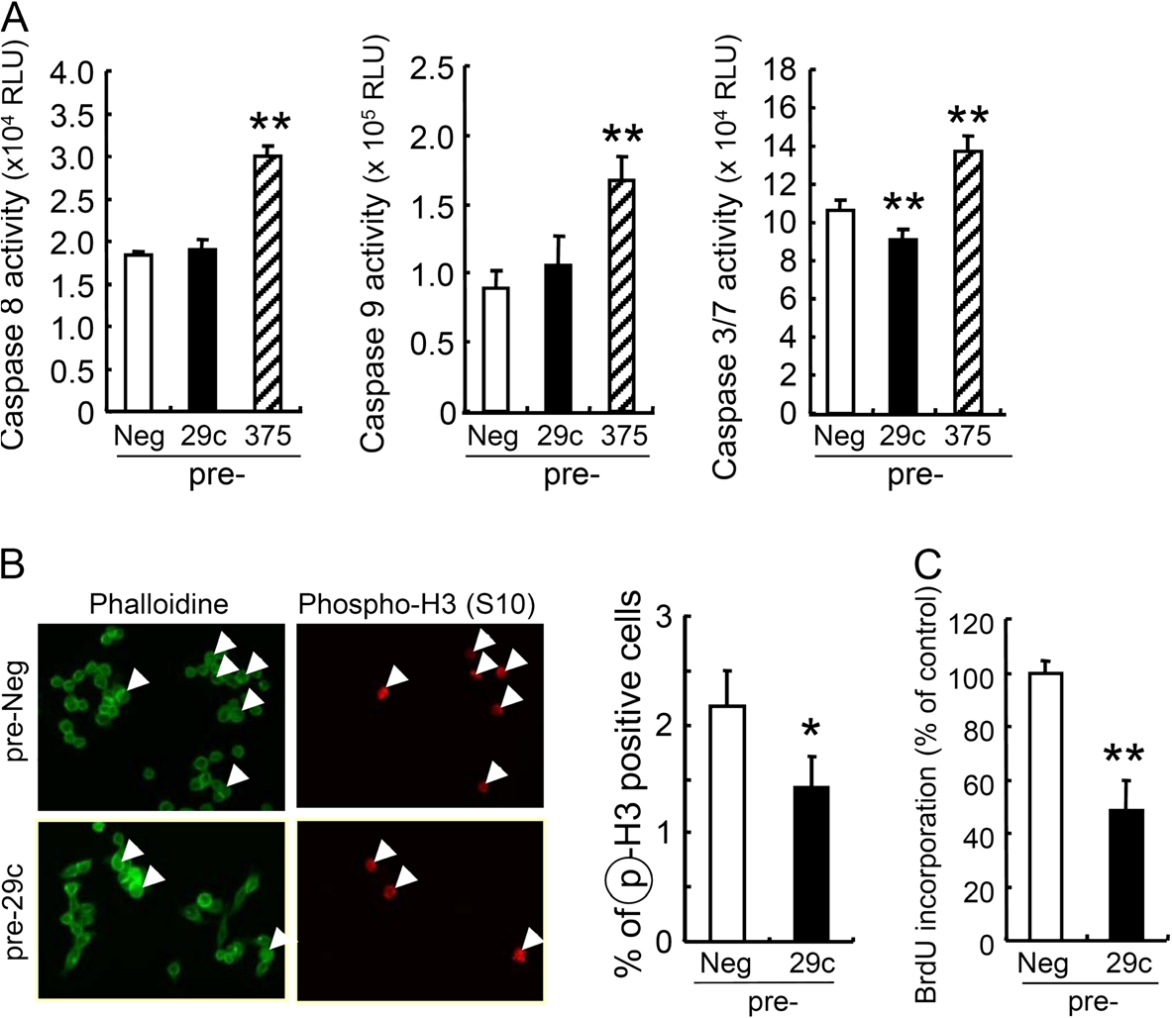
**miR-29c induces growth inhibition, but not apoptosis.** MKN45 cells were transfected with pre-29c or pre-Neg. (**A**) Activities of caspases-8, -9 and −3/7 were determined at 48 h after transfection. These data are shown as mean ± SD for three or four wells. miR-375 was used as a positive control. (**B**) Mitotic cells were labeled with phospho-histone H3 (Ser10) at 24 h. The proportion of positive cells was quantified by counting at least 500 cells in each of 3 or 4 wells, and is shown as a graph. (**C**) BrdU incorporation was determined by ELISA at 24 h. *P <0.05, **P <0.01.

To determine whether the growth inhibition by miR-29c was associated with apoptosis, caspase activities in MKN45 cells were determined at 48 h after transfection with pre-29c. We found that the activities of caspases-8, -9 and −3/7 were not increased in pre-29c-transfected cells (Figure 3A), while activation of caspases was observed in cells transfected with miR-375 (Figure 3A), similarly to our previous study [6]. This suggests that the growth suppression by miR-29c may be independent of apoptosis. Next, to examine whether miR-29c regulates the cell cycle, we calculated the proportion of the cells positive for phospho-histone H3 at Ser10, a marker of chromosome condensation during mitosis [12,13], using fluorescence immunocytochemistry at 24 h after transfection. As shown in Figure 3B, the positivity rate was significantly reduced in miR-29c transfected cells. Also, by measuring the incorporated BrdU, we determined the rate of DNA synthesis at 24 h after transfection, when the numbers of cells transfected with pre-Neg and miR-29c were not significantly different (Figure 2A). As shown in Figure 3C, BrdU incorporation was significantly reduced after pre-29c transfection. Similarly, in MKN74 and MKN 7 cells, pre-29c transfection also suppressed BrdU incorporation, but did not induce caspase-3/7 activity (data not shown). These results indicate that exogenous miR-29c causes growth suppression in gastric carcinoma cells by inhibition of the cell cycle, but does not induce apoptosis. During preparation of this manuscript, Saito Y and colleagues reported that overexpression of miR-29c induced apoptosis in MKN45 cells [14]. The difference in the results between that study and the present one may have been due to differences in the experimental conditions employed, such as the concentrations of oligonucleotides used for transfection (100 nM in [14]) and the transfection reagent (Oligofectamine in [14]).

### MiR-29c regulates the expression of RCC2, PPIC and CDK6 in gastric carcinoma cells

**Figure 4:**
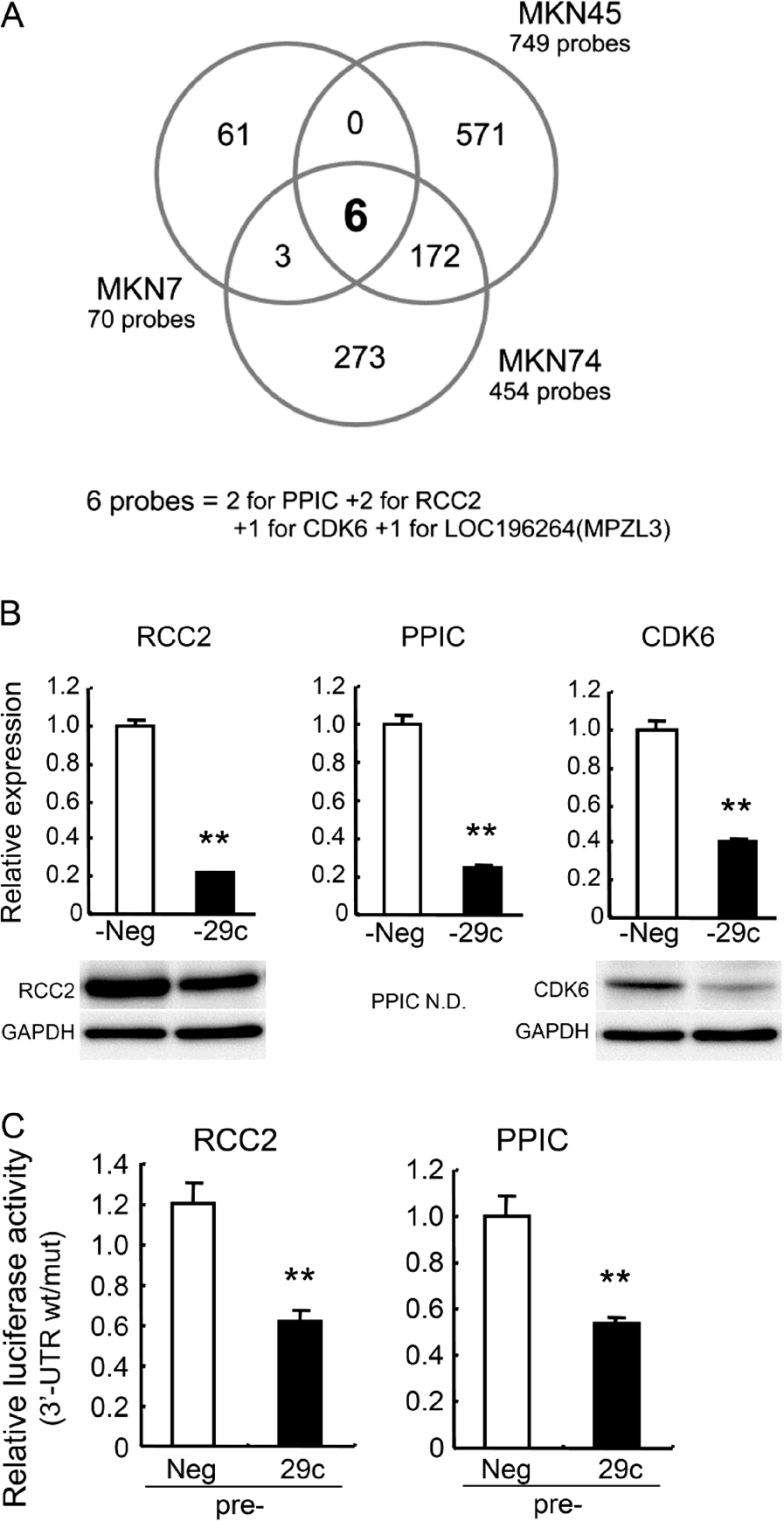
**miR-29c directly targets RCC2, PPIC and CDK6.** (**A**) Gene-expression microarray was performed at 24 h after transfection with pre-29c or pre-Neg. The Venn diagram shows the relationship between the sets of genes differentially expressed in MKN45, MKN74 and MKN7 cells. Changes in the expression of 4 genes (6 probes) were common to three cell lines. In the 3’-UTRs of PPIC, RCC2 and CDK6 mRNAs, the target sequences of miR-29c were found (according to TargetScan). (**B**) MKN45 cells were transfected with pre-29c or pre-Neg. The mRNA and protein levels of RCC2, PPIC and CDK6 were determined by quantitative RT-PCR and immunoblotting at 72 h. The mRNA levels relative to negative controls are indicated as mean ± SD of triplicate determinations. PPIC protein was not detectable (N.D.), presumably because the specificity of the PPIC antibody was insufficient. (**C**) Interaction of miR-29c with the 3’UTR of RCC2 and PPIC. At 24 h after transfection with pre-29c or pre-Neg, a reporter plasmid containing RCC2 or PPIC wt-3’UTR or mut-3’UTR and a plasmid expressing renilla luciferase (pRL-CMV) were cotransfected into MKN45 cells. Luciferase activities were measured at 48 h after transfection with the plasmids, and normalized data are shown. The data are indicated as mean ± SD of quadruplicate determinations. Three independent experiments were performed and representative data are shown. **P <0.01.

To investigate the mechanism of cell cycle inhibition by miR-29c, we performed expression microarray analysis of MKN45, MKN7 and MKN74 cells transfected with pre-29c or pre-Neg. At 24 h after transfection, 749 probes were differentially expressed by >2-fold in pre-29c-transfected MKN45 cells relative to pre-Neg-transfected cells, and 454 probes and 70 probes were differentially expressed in MKN74 cells and MKN7 cells, respectively (Figure 4A). It was noteworthy that only 6 probes for 4 genes (CDK6, RCC2, PPIC and MPZL3) were shared among three comparisons, and that in addition, their signals were reduced in pre-29c-transfected cells in all 3 comparisons. Three of the four genes, CDK6, RCC2 and PPIC, whose 3’-UTR of mRNAs possessed the miR-29c binding sequence, were identified as possible targets of miR-29c using the TargetScan algorithm (http://www.targetscan.org/). In fact, these three genes were downregulated at both the mRNA and protein levels in pre-29c-transfected MKN45 cells (Figure 4B), suggesting that miR-29c can regulate the expression levels of CDK6, RCC2 and PPIC in gastric carcinoma cells.

It has been demonstrated that miR-29c directly targets the 3’UTR of CDK6 mRNA and suppresses its expression at both the mRNA and protein levels [15], whereas the relationship between miR-29c and two other candidates, RCC2 and PPIC, has not been reported. Therefore we examined whether miR-29c is able to interact directly with the 3’UTR of RCC2 and/or PPIC. The miR-29c binding sequence at the 3’UTR of RCC2 mRNA (RCC2wt-3’UTR) or its mutant (RCCmut-3’UTR) was cloned downstream of the firefly luciferase reporter gene, and then cotransfected with pre-29c or pre-Neg into MKN45 cells. When pre-29c was cotransfected, the relative luciferase activity of the reporter containing RCC2wt-3’UTR was significantly suppressed in comparison with that of the reporter containing RCC2mut-3’UTR (Figure 4C). In contrast, the luciferase activity of the reporter containing RCC2wt-3’UTR was unaffected by simultaneous transfection with pre-Neg (Figure 4C). Similar results were obtained using luciferase reporter plasmids containing the miR-29c binding sequence at the 3’UTR of the PPIC mRNA or its mutant (Figure 4C). These results indicate that miR-29c is able to regulate the expression of CDK6, RCC2 and PPIC by directly binding to their 3’UTRs.

### RCC2 and PPIC are significantly upregulated in gastric carcinoma tissues

**Figure 5:**
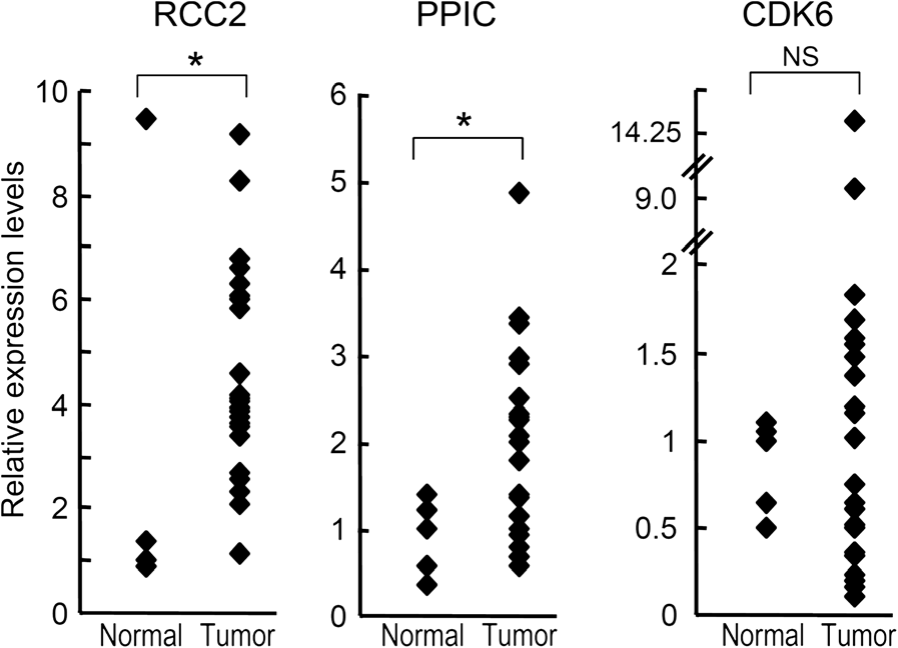
**RCC2 and PPIC, but not CDK6, are upregulated in gastric carcinoma.** To validate the expression levels of possible miR-29c target genes in cancer tissues, quantitative RT-PCR was performed with non-cancerous epithelial tissues (n = 5) and gastric carcinoma tissues (n = 23) that had been previously subjected to microarray [6,21]. The graphs show the normalized expression levels of individual miRNAs relative to the median of non-cancerous epithelial tissues. Each dot indicates the expression level in an individual case. The y axis represents the fold change. *P <0.05. NS, not significant. Mann–Whitney U test.

Since miR-29c was significantly downregulated in gastric carcinoma, its possible targets were expected to be upregulated. To clarify this issue, we analyzed the expression levels of RCC2, PPIC and CDK6 mRNAs in gastric carcinoma tissues and normal epithelial tissues using quantitative RT-PCR. As shown in Figure 5, the expression levels of RCC2 and PPIC were actually upregulated in carcinoma tissues relative to normal tissues, whereas CDK6 was not. This suggests that RCC2 and PPIC may be the target of miR-29c in gastric carcinoma, whereas CDK6 may not.

### RCC2 reduction potentially contributes to the growth suppression induced by miR-29c

**Figure 6:**
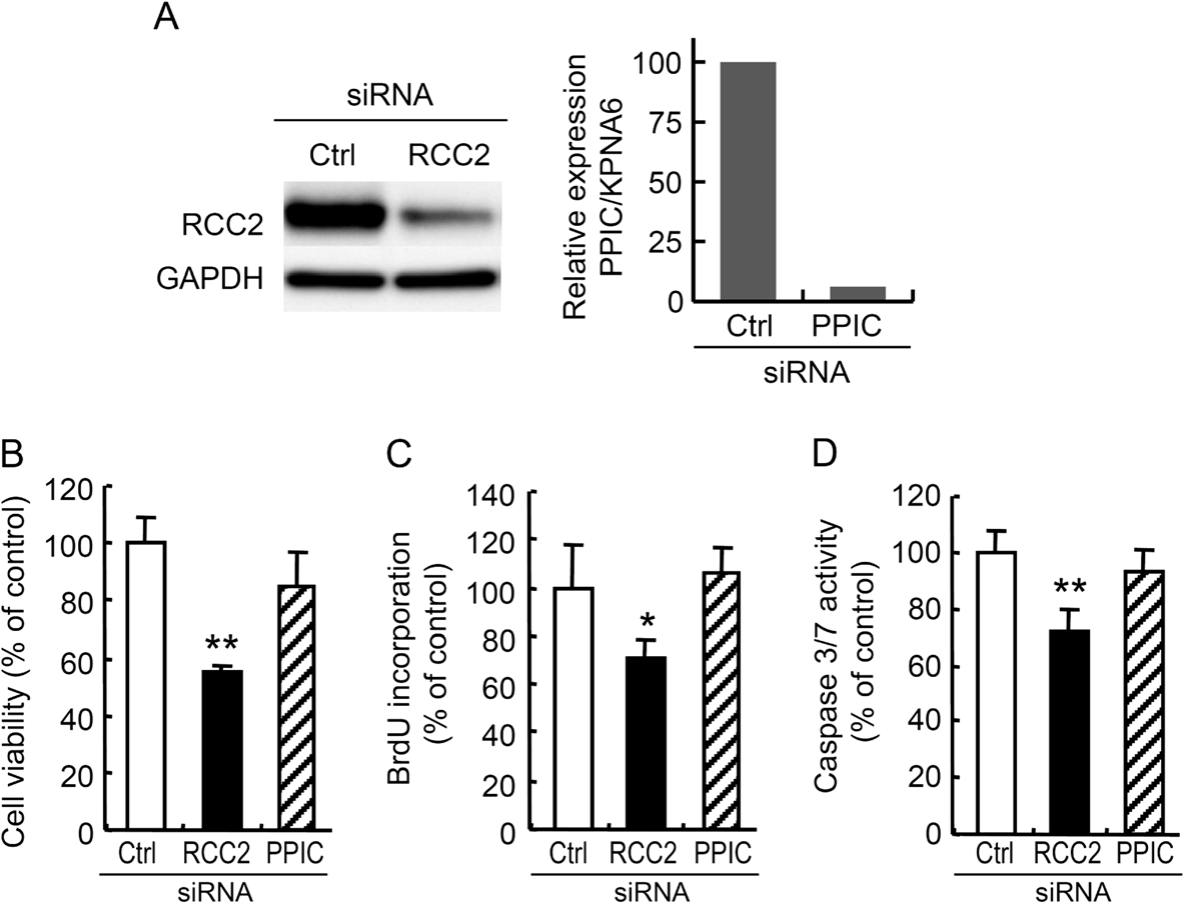
**RCC2 knockdown suppresses cell proliferation.** MKN45 cells were transfected with siRNA against RCC2, PPIC or a negative control. The downregulation of RCC2 protein and PPIC mRNA was confirmed (**A**). Cell viability at 72 h (**B**), BrdU incorporation at 24 h (**C**) and caspase-3/7 activities at 72 h (**D**) were determined. These data are shown as mean ± SD of quadruplicate determinations. *P <0.05. **P <0.01.

To assess the contribution of RCC2 and PPIC to the growth suppression by miR-29c, we transfected siRNAs against RCC2 and PPIC into MKN45 cells and measured the resulting cell viabilities by MTS assay at day 3. Downregulation of RCC2 at the protein level and PPIC at the mRNA level was confirmed by immunoblotting and quantitative RT-PCR, respectively (Figure 6A). As shown in Figure 6B, cell viability was significantly decreased only in RCC2 siRNA-transfected cells, and not in PPIC siRNA-transfected cells. We also measured BrdU incorporation and found that it was significantly reduced only in RCC2 siRNA-transfected cells (Figure 6C). In addition, caspase-3/7 was not activated in RCC2 siRNA-transfected cells (Figure 6D) or in pre-29c-transfected cells (Figure 3A), indicating that these phenotypes resulting from transfection with RCC2 siRNA closely resembled those resulting from pre-29c transfection. Taken together, these findings suggested that downregulation of RCC2 may be at least partly involved in the growth suppression induced by miR-29c. On the other hand, cells transfected with PPIC siRNA did not exhibit growth suppression (Figure 6B and 6C), although its mRNA level was decreased (Figure 6A), suggesting that PPIC may not be involved in the regulation of cell proliferation. However, we were unable to exclude the possibility that PPIC siRNA failed to suppress the expression of PPIC at the protein level.

## Discussion

MiR-29c has been reported to be downregulated in various types of tumors [16-19], including gastric carcinoma [6,20]. In nasopharyngeal carcinomas, miR-29c reportedly regulates the expressions of extracellular matrix proteins [17], implicating the involvement of miR-29c downregulation in tumor invasiveness. In esophageal squamous cell carcinoma, miR-29c has been shown to induce cell cycle arrest by regulating the expression of cyclin E [16]. However, in gastric carcinoma, there have been no data to suggest the function and targets of miR-29c. In the present study, we showed that ectopic expression of miR-29c suppressed the proliferation of gastric carcinoma cells and their ability to form colonies on soft agar. Furthermore, the growth suppression was not accompanied by caspase activation, but occurred through a decrease in the rate of DNA synthesis, suggesting that miR-29c may suppress cell proliferation by regulating progression of the cell cycle. To our knowledge, this is the first report to describe the tumor-suppressive role of miR-29c in gastric carcinoma. The mechanism responsible for miR-29c downregulation in gastric carcinoma is still unknown. Since genomic loss at 1q32.2, where miR-29c is located, is rarely detected in gastric carcinoma, whereas 1q gain is frequently observed [21], it is unlikely that genomic copy number alteration is responsible for the downregulation of miR-29c. In addition, we showed that miR-29c downregulation is probably not associated with DNA methylation or histone deacetylation. Other factors, such as interaction of transcriptional suppressors with the miR-29c promoter and post-transcriptional regulation may play a role. Further studies will be needed to clarify these issues.

In this study, we showed that miR-29c reduced the expression of RCC2 at both the mRNA and protein levels. The luciferase assay with a reporter containing the miR-29c binding sequence at the 3’UTR of RCC2 mRNA suggested that miR-29c directly targets the 3’UTR of RCC2 mRNA. Although RCC2 (also known as TD-60) is reportedly a component of the chromosomal passenger complex, which is a crucial regulator of chromosomes, the cytoskeleton, and membrane dynamics throughout mitosis [22,23], little has been known about the oncogenic relevance of RCC2. Mollinari and colleagues showed that RCC2 knockdown induced cell cycle arrest at prometaphase in HeLa cells due to failure of spindle assembly, suggesting that RCC2 regulates cell cycle progression by modulating chromosome segregation and cell cleavage [24]. In this study, we showed for the first time that RCC2 is upregulated in gastric carcinoma tissues. Furthermore, knockdown of RCC2 induced growth suppression without caspase activation in gastric carcinoma cells. Together with the data reported previously by other groups, our results suggest that RCC2 contributes to the proliferation of gastric carcinoma cells by regulating progression of the cell cycle. Interestingly, growth suppression without caspase activation was also observed in miR-29c-transfected cells, suggesting that downregulation of RCC2 may be involved in the miR-29c-induced growth suppression. In addition, both miR-29c and RCC2 transfection decreased cell viability accompanied by a reduction of BrdU incorporation, suggesting that RCC2 may play a role in/around S phase of the cell cycle. Thus, our data suggest that miR-29c reduces the expression of RCC2 in gastric carcinoma cells, leading to suppression of their growth, not through induction of apoptosis but by a cell cycle regulation signal. Therefore, we can hypothesize that decreased expression of miR-29c results in enhanced expression of RCC2, and confers a growth advantage on gastric carcinoma cells. Because the contribution of RCC2 to cell cycle regulation signaling during S phase of the cell cycle is still poorly understood, further studies will be needed to clarify this issue.

On the other hand, PPIC siRNA-transfected cells did not exhibit growth suppression, although PPIC mRNA level was decreased. We demonstrated that PPIC is one of the target genes of miR-29c and was significantly upregulated in gastric cancer tissues. Therefore, PPIC may play some role as a downstream molecule of miR-29c, although it may not be involved in cell proliferation and apoptosis. It has been reported that PPIC, also known as cyclophilin C, is a cellular binding protein for the immunosuppressive drug cyclosporine A, which can suppress T-cell activation [25-27]. Moreover, it has been reported that the natural cellular ligand for PPIC, cyclophilin C-associated protein (CyCAP, also kinown as MAC2BP) [28], can act as a modulator of endotoxin signaling in vivo [29]. These facts led us to speculate that aberrant expression of PPIC may affect some aspects of the immune response, such as inflammation, during gastric carcinogenesis. However, to assess the function of PPIC under miR-29c regulation, further studies will be needed.

In gastric carcinoma tissues, CDK6 upregulation was not observed, at least at the mRNA level, although CDK6 expression was suppressed at both the mRNA and protein levels by ectopic expression of miR-29c in MKN45 cells. These findings suggest that CDK6 expression in tissue is probably affected by other factors. Indeed, upregulation of CDK6 in gastric carcinoma has been reported by other research groups [30,31], and a portion of cases showing CDK6 overexpression harbored chromosomal amplification of 7q21.2, where the CDK6 gene is located [31].

## Conclusions

We have found that miR-29c was downregulated in a substantial proportion of gastric carcinomas and suppressed proliferation of gastric carcinoma cells, potentially by targeting RCC2. Furthermore, miR-29c reduced the ability of gastric carcinoma cells to form colonies on soft agar. Therefore, we propose that miR-29c may have a tumor-suppressive role in gastric carcinoma cells, and that its decreased expression may confer a growth advantage on tumor cells at least partly via aberrant expression of RCC2, one of the target genes of miR-29c. Future studies examining the mechanism of miR-29c downregulation and the function of RCC2 in cell cycle regulation will be required in order to clarify the significance of miR-29c-RCC2-dependent cell cycle regulation in gastric carcinoma cells.

## Materials and methods

### Tissues

Gastric carcinoma tissues were surgically resected from 12 patients at Oita University Hospital. Information on the patients is summarized in Additional file 1. The tumor samples were fixed in 10% buffered formalin, and then embedded in paraffin. Use of the tissue samples for all experiments was approved by all of the patients and by Oita University Ethics Committee.

### Cell culture and transfection

The gastric carcinoma cell lines MKN45, MKN74 and MKN7 were purchased from JCRB, and maintained in RPMI supplemented with 10% FCS. The cells were transfected with 10 nM small RNAs (see below) using Lipofectamine RNAiMAX (Invitrogen) and with plasmids using X-tremeGENE 9 (Roche Diagnostics) in accordance with the manufacturer’s instructions.

Small RNAs: pre-miR-29c precursor miRNA and negative control precursor miRNA (Ambion), RCC2 and PPIC siRNA ON-TARGETplus SMARTpool and negative control pool (Dharmacon).

### MTS assay, BrdU ELISA and caspase activity

Cells were transfected with RNA oligos, and cell viability was measured using the CellTiter96 aqueous one solution cell proliferation assay (Promega) after the indicated periods. The rate of DNA synthesis was determined 24 h after transfection, at a time point when significant differences in cell number by MTS assay were not observed between groups, using a Cell Proliferation ELISA BrdU kit (Roche) in accordance with the manufacturer’s instructions. Activities of caspases-3/7, -8 and −9 were measured using the Caspase-Glo assay (Promega) in accordance with the manufacturer’s instructions. Statistical comparisons were performed using unpaired Student’s *t* test.

### 5-aza-dC and TSA treatment

For treatment with 5-aza-2’-deoxycytidine (5-aza-dC; Sigma-Aldrich), MKN45 cells (8 x10^4^) were seeded into 35-mm dishes on day 0 and exposed to 5 μmol/L 5-aza-dC from day 1 to day 4. Trichostatin A (TSA; Sigma-Aldrich) was added into the cells on day 3 at a concentration of 100 nmol/L. The cells treated with either 5-aza-dC, TSA, or both were harvested on day 4. Two independent experiments were performed (Additional file 2).

### RNA extraction

Tissue sections (10 μm thick) from formalin-fixed, paraffin-embedded samples were stained with toluidine blue (Wako, Japan). Tumor cells and normal cells were collected separately under microscopic observation. Extraction of total RNA containing miRNA was performed using a RNeasy FFPE kit (QIAGEN) in accordance with the manufacturer’s instructions for miRNA containing total RNA.

Total RNA from cell lines was extracted with a miRNeasy Mini kit (QIAGEN) for quantitive RT-PCR, whereas total RNA for gene-expression microarray was extracted with a RNeasy Mini kit (QIAGEN) in accordance with the manufacturer’s instructions.

### Quantitative RT-PCR

Quantitative RT-PCR for miRNA and mRNA was performed in the same way as for our previous work (standard curve, Taqman method) [6,32]. Levels of miRNA expression were determined relative to RNU44 and those of mRNA expression were determined relative to KPNA6.

### Soft agar colony formation assay

Two or three days after transfection, cells were subjected to soft agar assays using 96-well plates containing semi-solid medium (10% FBS-DMEM, 0.4% agar) with 2.3 × 10^3^ (MKN45) and 2 × 10^3^ (MKN74) cells/well on a base layer of medium (10% FBS-DMEM, 0.6% agar). The cells were grown for 8–9 days, and the colonies were observed using a light microscope. To quantify the efficiency of colony formation, the CytoSelect™ 96-Well Cell Transformation Assay (Cell Biolabs) was used in accordance with the manufacturer’s instructions. Three independent experiments were performed.

### Antibodies

Anti-CDK6 (Cell Signaling Technology) and anti-GAPDH mouse monoclonal antibodies (Ambion), and anti-RCC2 (Cell Signaling Technology) rabbit polyclonal antibody were used as primary antibodies. GAPDH was detected as an internal control by immunoblotting.

### Immunocytochemistry

Cells were fixed with 4% paraformaldehyde for 10 min at RT. After washing with PBS, the cells were permeabilized in 0.3% Triton X-100/PBS for 10 min. The cells were then preincubated in 2% BSA/PBS for 30 min, followed by incubation for 2 h with anti-phospho-histone H3 (S10) (6 G3) antibody (Cell Signaling Technology). After washing with 0.1% Triton X-100/PBS, the cells were incubated with Alexa568-conjugated secondary antibody for 1 h. After a further wash, the cells were counterstained with Alexa488-conjugated phalloidin. The mounted cells were observed with a fluorescence microscope (Leica).

### Gene-expression microarray

Three hundred nanograms of total RNA was subjected to microarray analysis in a similar way to that described in our previous study [6,21]. Briefly, Cy3-labeled cRNA was generated using a Quick Amp labeling kit (Agilent Technologies) and then hybridized to a human 44 K oligoarray (G4112F#014850, Agilent Technologies). Microarray images were obtained using a laser confocal scanner G2565BA (Agilent Technologies) and analyzed using Feature Extraction v.9.5.3.1 (Agilent Technologies) with the manufacturer’s recommended settings. The resulting data were subsequently imported into GeneSpring GX10 software (Agilent Technologies). For comparisons among multiple arrays, probe set data were median-normalized per chip. Then, the data were centered across the genes, followed by filtering based on signal intensity and flagged values. Differentially expressed genes were identified using a filter based on a fold-change of 2.0. All data are available at GEO via NCBI under Accession No.GSE38581 (http://www.ncbi.nlm.nih.gov/geo/query/acc.cgi?acc = GSE38581).

### Luciferase reporter assay

Reporter plasmids were constructed as described previously, with modifications [6]. Double-stranded oligonucleotides corresponding to the wild-type (wt-3’UTR) or mutant (mut-3’UTR) miR-29c binding site in the RCC2 3’UTR or PPIC 3’UTR were synthesized and ligated between the SpeI and HindIII restriction sites of the reporter plasmid pMIR-Report (Ambion). The oligonucleotides used are described in Additional file 3. At 24 h after transfection with pre-29c or pre-Neg, a reporter plasmid containing the wt-3’UTR or mut-3’UTR and a plasmid expressing *Renilla* luciferase (pRL-CMV) (Promega) were co-transfected into MKN45 cells. Firefly luciferase activity derived from pMIR-Report was normalized to *Renilla* luciferase activity from pRL-CMV. Normalized luciferase activity in cells transfected with wt-3’UTR was compared with that in cells transfected with mut-3’UTR.

## Consent

Written informed consent was obtained from the patient for publication of this report and any accompanying images.

## Abbreviations

RCC2: Regulator of chromosome condensation 2 (also known as TD-60), NCBI Gene ID: 55920; PPIC: Peptidylprolyl isomerase C (also known as cyclophilin C), NCBI Gene ID: 5480; CDK6: Cyclin-dependent kinase 6, NCBI Gene ID: 1021; pre-29c: precursor miR-29c; pre-Neg: Negative control of precursor miRNA.

## Competing interests

The authors have no conflict of interest.

## Authors’ contribution

MiM, CN, YT and MaM conceived experiment and wrote the paper. MiM, CN and YT carried out experiments. All authors analyzed data. All authors had final approval of the submitted and published versions.

The raw and processed data from microarray analysis are available at GEO via NCBI under Accession No.GSE38581 (http://www.ncbi.nlm.nih.gov/geo/query/acc.cgi?acc = GSE38581).

## Supplementary Material

Additional file 1 **Supplementary information 1.** Characteristics of the tumor samples. Click here for file

Additional file 2: Figure S1 Epigenetic modification may not be associated with miR-29c downregulation. Click here for file

Additional file 3 **Supplementary information 2.** The oligonucleotides used for construction of luciferase reporter plasmids. Click here for file
