# Supplementary material for: MiR-29c is downregulated in gastric carcinomas and regulates cell proliferation by targeting RCC2
Source: Mol Cancer. 2013 Feb 25;12:15. doi: 10.1186/1476-4598-12-15 (PMC3646694; doi:10.1186/1476-4598-12-15)
Supplement: Additional file 1 — Supplementary information 1. Characteristics of the tumor samples. [file 1476-4598-12-15-S1.docx]

| **Supplementary information 1. Characteristics of tumor samples.** | | | | | | | | |  |  |
| --- | --- | --- | --- | --- | --- | --- | --- | --- | --- | --- |
|  |  |  |  | |  | |  | |  |  |
| ID^a^ | Age/Sex | Type^b^ | Location^c^ | | Depth^c^ | | Stage^c^ | | miR-29c FC^d^ |  |
| case 6 | 70/M | intestinal | Corpus | | T4 | | IIIB | | 0.75 |  |
| case 14 | 77/M | intestinal | Fundus | | T2 | | IB | | 0.22 |  |
| case 15 | 74/F | intestinal | Antrum | | T3 | | IB | | 0.35 |  |
| case 5 | 76/F | intestinal | Fundus | | T3 | | IIIA | | 0.37 |  |
| case 17 | 77/F | intestinal | Corpus | | T4 | | IIIB | | 0.45 |  |
| case 18 | 65/M | intestinal | Fundus | | T3 | | IIIB | | 0.49 |  |
| case 19 | 58/M | intestinal | Fundus | | T4 | | IV | | 0.15 |  |
| case 2 | 75/M | diffuse | Corpus | | T4 | | IIIA | | 0.19 |  |
| case 3 | 74/M | diffuse | Corpus | | T4 | | IIIA | | 0.62 |  |
| case 8 | 88/F | diffuse | Corpus | | T4 | | IV | | 0.19 |  |
| case 10 | 79/M | diffuse | Antrum | | T3 | | IIIA | | 0.53 |  |
| case 13 | 40/F | diffuse | Corpus | | T4 | | IV | | 0.37 |  |
|  |  |  |  | |  | |  | |  |  |
| ^a^ These cases are shared with our previous study. | | | | |  | |  | |  |  |
| Thus these IDs correspond to those in our previous reports | | | | | | |  | |  |  |
| [21] Tsukamoto et al., J Pathol 2008 and [6] Tsukamoto et al., Cancer Res 2010). | | | | | | | | |  |  |
| ^b^ According to Lauren classification. | | | |  | |  | |  |  |  |
| ^c^ According to the criteria of Japanese Classification of Gastric Cancer. | | | | | | | | |  |  |
| ^d^ The fold change of miR-29c expression levels in tumor relative to normal tissue were presented (see Figure 1B). | | | | | | | | | | |
