# Supplementary figures and images for: MiR-29c is downregulated in gastric carcinomas and regulates cell proliferation by targeting RCC2
Source: Mol Cancer. 2013 Feb 25;12:15. doi: 10.1186/1476-4598-12-15 (PMC3646694; doi:10.1186/1476-4598-12-15)

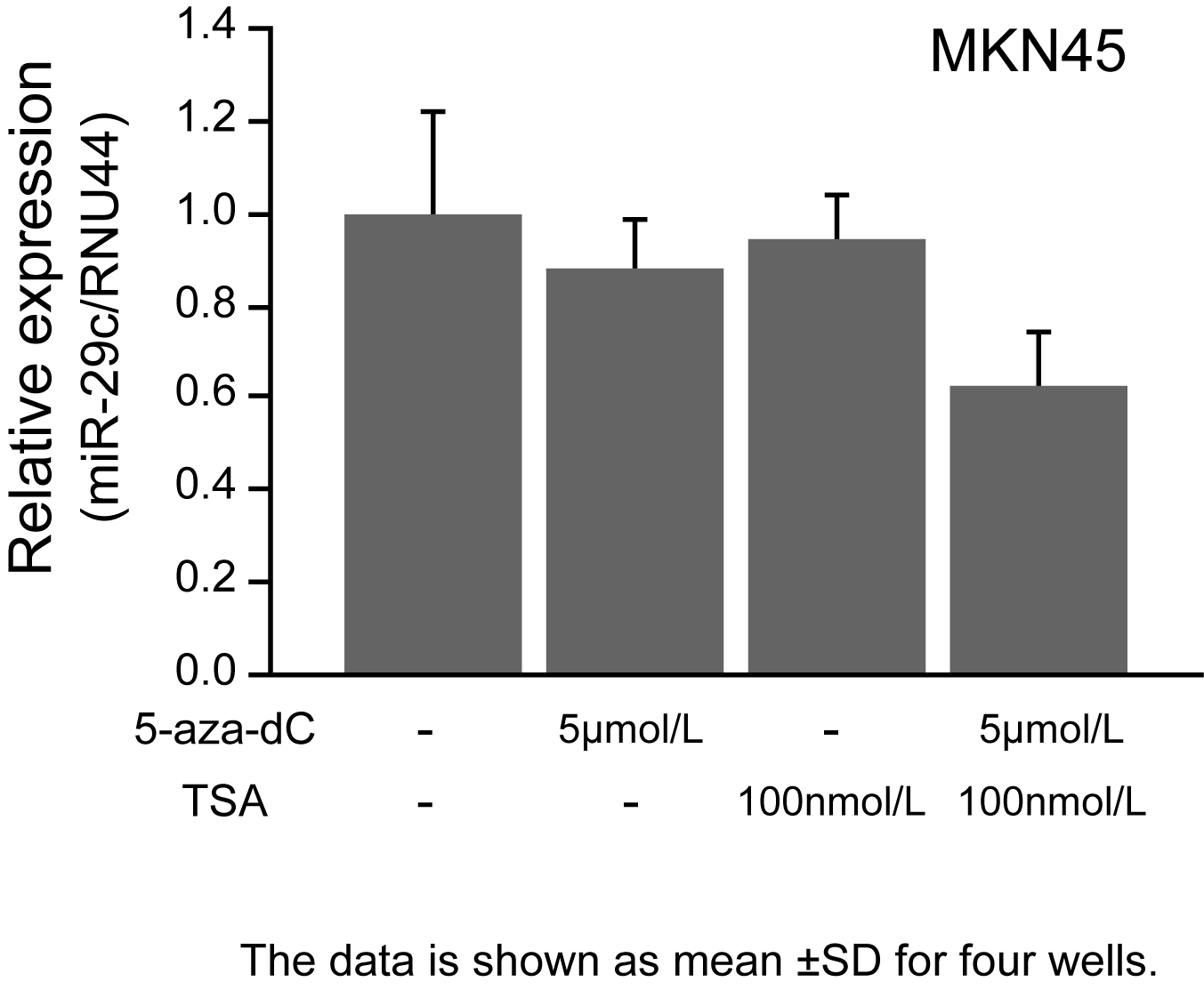

Supplement: Additional file 2: Figure S1 — Epigenetic modification may not be associated with miR-29c downregulation. [file 1476-4598-12-15-S2.tiff]
