# Supplementary material for: MiR-29c is downregulated in gastric carcinomas and regulates cell proliferation by targeting RCC2
Source: Mol Cancer. 2013 Feb 25;12:15. doi: 10.1186/1476-4598-12-15 (PMC3646694; doi:10.1186/1476-4598-12-15)
Supplement: Additional file 3 — Supplementary information 2. The oligonucleotides used for construction of luciferase reporter plasmids. [file 1476-4598-12-15-S3.doc]

**Supplementary information 2.**

**The oligonucleotides used for construction of luciferase reporter plasmids.**

RCC2wt F 5’-**CTAGT**AAATAGATATTTAGTTCAGTGGTGCTTATGGATCCA**A**

RCC2wt R 5’-**AGCTT**TGGATCCATAAGCACCACTGAACTAAATATCTATTT**A**

RCC2mut F 5’-**CTAGT**AAATAGATATTTAGTTCAGACCACGATATGGATCCA**A**

RCC2mut R 5’-**AGCTT**TGGATCCATATCGTGGTCTGAACTAAATATCTATTT**A**

PPICwt F 5’-**CTAGT**ATATCCCCTTCCTCAAGTGGTGCTATTTGGATCCA**A**

PPICwt R 5’-**AGCTT**TGGATCCAAATAGCACCACTTGAGGAAGGGGATAT**A**

PPICmut F 5’-**CTAGT**ATATCCCCTTCCTCAAGACCACGAATTTGGATCCA**A**

PPICmut R 5’-**AGCTT**TGGATCCAAATTCGTGGTCTTGAGGAAGGGGATAT**A**

Underlines, miR-29c target site

Bold and shade, *Spe I* site

Bold only, *Hind III* site

Shade only, *BamHI* site

The pairs of oligonucleotides were annealed and cloned into pMIR-report luciferase plasmid (Ambion).

*Spe I* and *HindIII* site were used for cloning, and *BamHI* site was used for insert check.
